# Supplementary material for: Paired associated SARS-CoV-2 spike variable positions: a network analysis approach to emerging variants
Source: mSystems. 2023 Jul 11;8(4):e00440-23. doi: 10.1128/msystems.00440-23 (PMC10469592; doi:10.1128/msystems.00440-23)
Supplement: Fig. S1 — Sampling distributions. [file msystems.00440-23-s0004.docx]

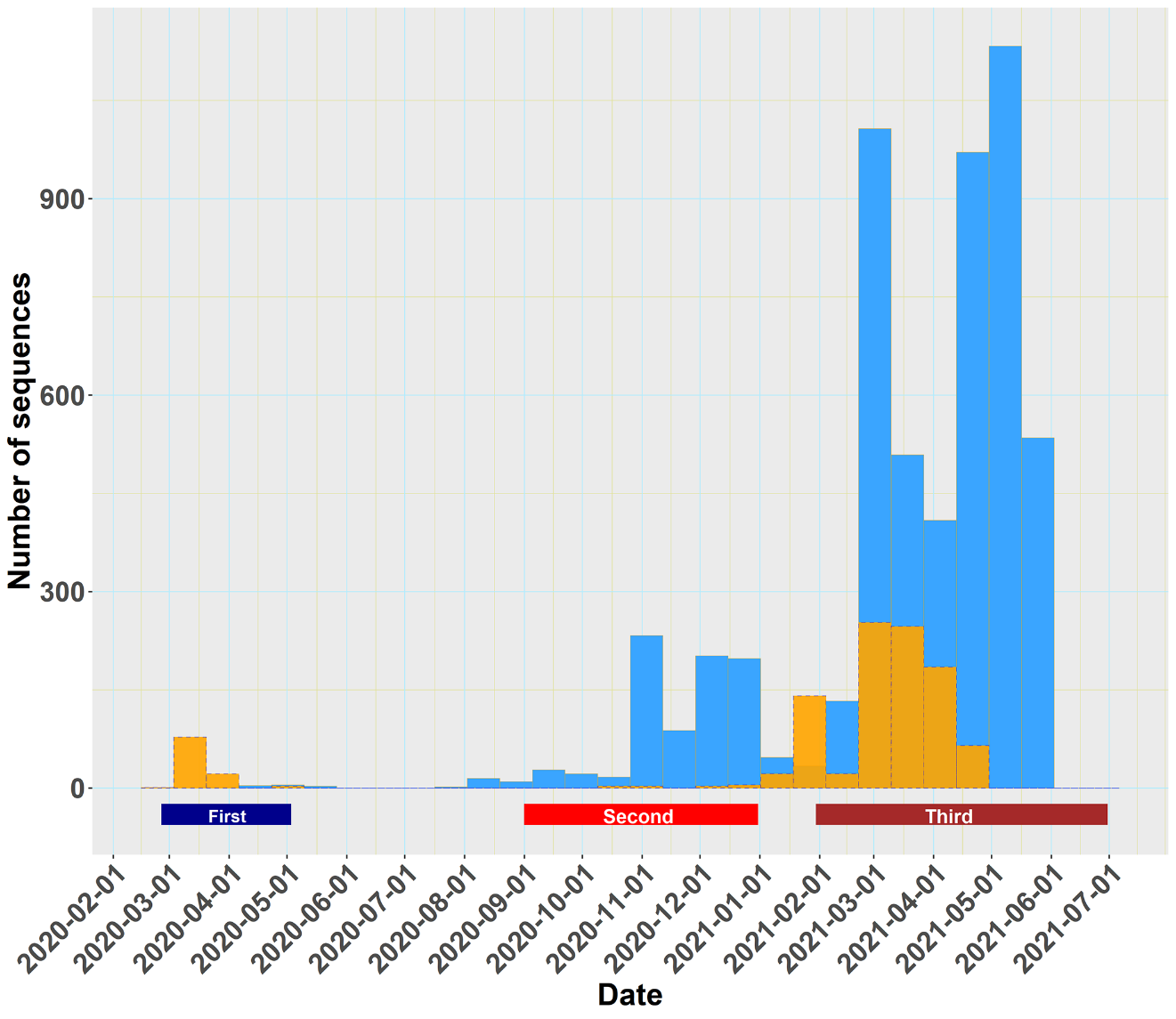


**SI Figure 1.** Distribution in time of whole genome (orange) and partial genome (spike only, cyan) sequences used for network construction and mutational frequency analysis. Labels indicate the first, second and third pandemic waves.
