# Supplementary material for: Paired associated SARS-CoV-2 spike variable positions: a network analysis approach to emerging variants
Source: mSystems. 2023 Jul 11;8(4):e00440-23. doi: 10.1128/msystems.00440-23 (PMC10469592; doi:10.1128/msystems.00440-23)
Supplement: Fig. S2 — Sampling by region. [file msystems.00440-23-s0005.docx]

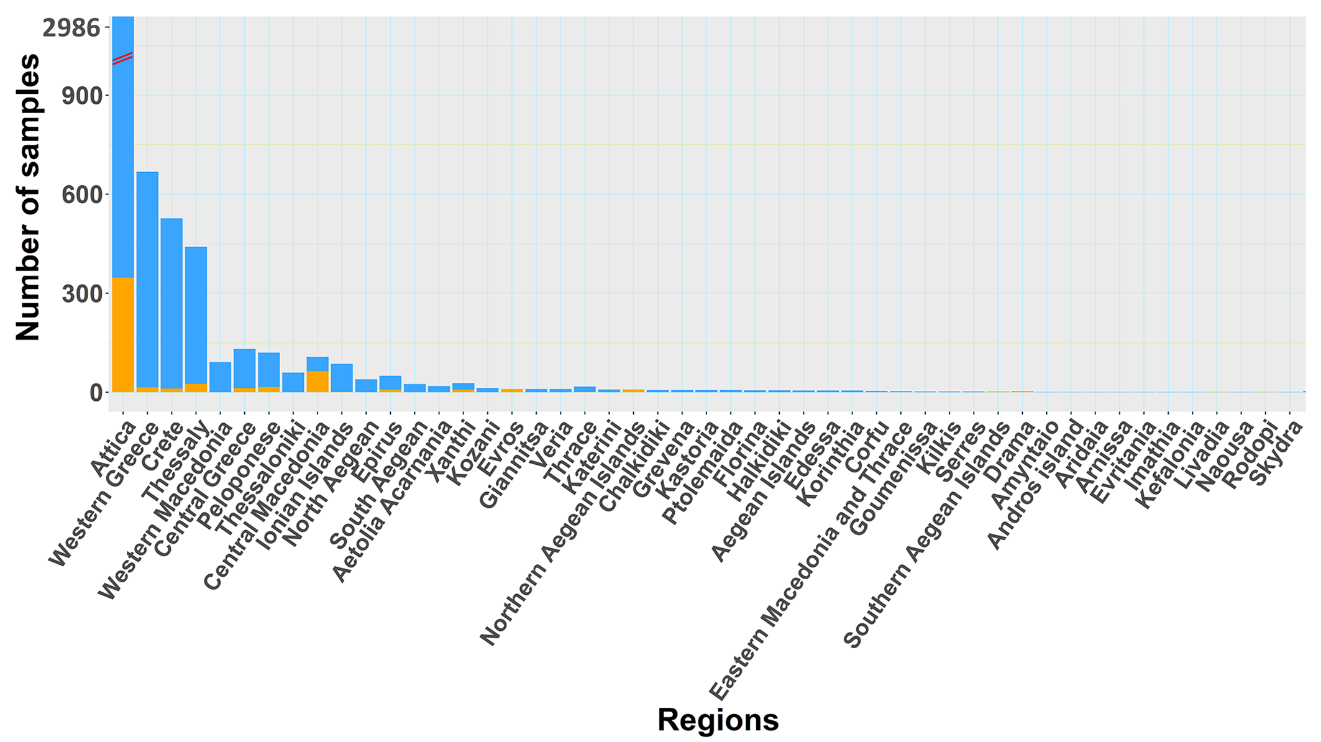


**SI Figure 2.** Stacked bar plot showing regional distribution of whole genome (orange) and partial genome (spike only, cyan) sequences used for network construction and mutational frequency analysis.
