# Supplementary material for: Paired associated SARS-CoV-2 spike variable positions: a network analysis approach to emerging variants
Source: mSystems. 2023 Jul 11;8(4):e00440-23. doi: 10.1128/msystems.00440-23 (PMC10469592; doi:10.1128/msystems.00440-23)
Supplement: Fig. S3 — Probabilities of identified variable spike protein positions (n=170) related to time. [file msystems.00440-23-s0006.docx]

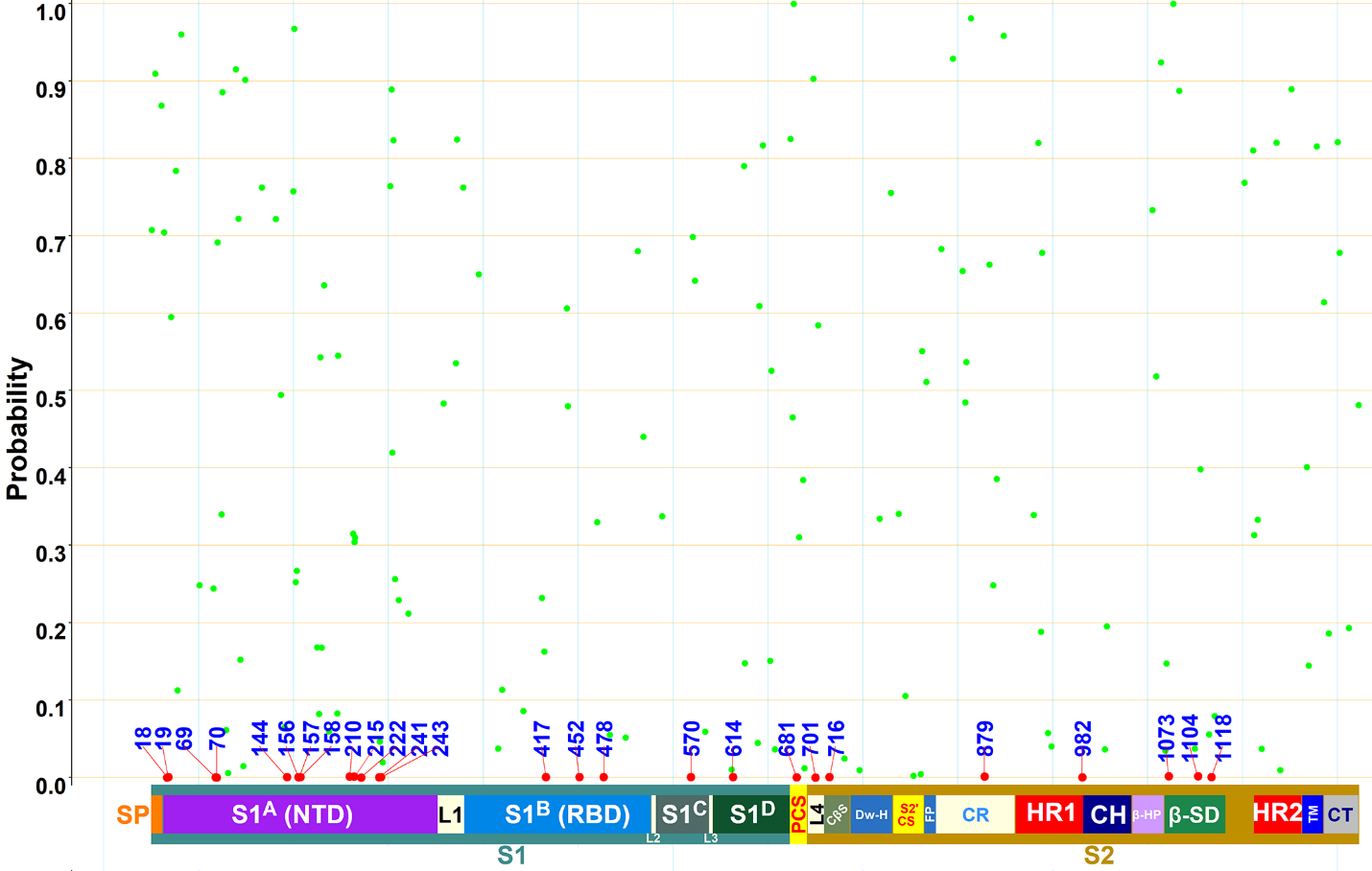


**SI Figure 3.** Probabilities of identified variable spike protein positions (n=170) related to time. Green dots indicate probability values >0.001 of the Day variable. Numbers with dots indicate positions. Red color indicates significances (p≤0.001) for the corresponding positions. The map represents the spike protein. The dark green and brown bars display the S1 and S2 regions, respectively. SP= signal peptide, NTD=N’ terminal domain, RBD = Receptor binding domain, HR=Heptad Repeat (1 and 2).
