# Supplementary material for: Paired associated SARS-CoV-2 spike variable positions: a network analysis approach to emerging variants
Source: mSystems. 2023 Jul 11;8(4):e00440-23. doi: 10.1128/msystems.00440-23 (PMC10469592; doi:10.1128/msystems.00440-23)
Supplement: Table S1 — Selected SARS-CoV-2 variant-defining spike amino acid signatures. [file msystems.00440-23-s0007.docx]

**SI Table 1**. Selected SARS-CoV-2 variant-defining spike amino acid signatures.

| **Variant** | **Amino acid signatures in spike** |
| --- | --- |
| **Alpha (B.1.1.7)** | del69-70, del144, 501Y, 570D, 614G, 681H, 716I, 982A, 1118H |
| **Beta (B.1.351)** | 80A, 215G, Del241-243, 417N, 484K, 501Y, 614G, 701V |
| **Gamma (P.1)** | 18F, 20N, 26S, 138Y, 190S, 417N/T, 484K, 501Y, 614G, 655Y, 1027I, 1176F |
| **Delta (21A, B.1.617.2)** | 19R, 142D, del156-157, 158G, 452R, 478K, 614G, 681R, 950N |
| **Epsilon (B.1.427, B.1.429, CAL.20C)** | 13I, 152C, 452R, 614G |
| **Zeta (P.2, B.1.1.28.2)** | 484K, 614G, 656L, 859I, 1176F |
| **Eta (B.1.525, 20A/S:484K)** | 52R, 67V, del69-70, del144, 484K, 614G, 677H, 888L |
| **Theta (P.3, B.1.1.28.3, 21E)** | 484K, N501Y, 614G, P681H, 1092K, 1101Y, 1176F |
| **Iota (B.1.526, 21F)** | 5F, 95I, 253G, 477N, 484K, 614G, 701V |
| **Kappa (B.1.617.1, 20A/S:154K)** | 95I, 142D, 154K, 452R, 484Q, P681R, 1071H |
| **Lambda (C.37, B.1.1.1.C37)** | 75V, 76I, del246-252, 452Q, 490S, 614G, 859N |
| **B.1.1.318** | 95I, del144, 484K, 614G, 681H, 796H |
| **A.23.1** | 102I, 157L, 367F, 613H, 681R |
| **B.1.616** | 66D, del144/145, 215G, 483A, 655Y, 669S, 949R, 1187D |
| **B.1.214.2** | ins214TDR, 414K, 450K, 614G, 716I |
