# Supplementary material for: Paired associated SARS-CoV-2 spike variable positions: a network analysis approach to emerging variants
Source: mSystems. 2023 Jul 11;8(4):e00440-23. doi: 10.1128/msystems.00440-23 (PMC10469592; doi:10.1128/msystems.00440-23)
Supplement: Table S2 — Dates of first reports of SARS-CoV-2 index virus and variants in Greece. [file msystems.00440-23-s0008.docx]

**SI Table 2.** Dates of first reports of SARS-CoV-2 index virus and variants in Greece.

| **Variant** | **GISAID**  **accession number** | **Year** | **Month-Day** |
| --- | --- | --- | --- |
| Wuhan-Hu-1 | EPI_ISL_2232041 | 2020 | Feb 26 |
| Alpha | EPI_ISL_2301679 |  | Dec 11 |
| Beta | EPI_ISL_1716721 | 2021 | Jan 14 |
| Gamma | EPI_ISL_2454299 |  | May 14 |
| Delta-like* | EPI_ISL_1716736 |  | Mar 23 |
| Eta | EPI_ISL_1716722 |  | Mar 8 |
| Kappa | EPI_ISL_1716737 |  | Apr 6 |
| B.1.1.318 | EPI_ISL_2370918 |  | Feb 5 |

* Eight, presumably Delta precursor isolates, were detected as follows: EPI_ISL_1716736 and EPI_ISL_2364992, carrying 19R, 156G, Δ157-158, 452R, 478K, 614G, 681R and 950N, were collected on March 23, 2021 and April 28, 2021 respectively; four isolates, harboring 19R, 156G, Δ157-158, 452R, 478K, 614G, and 681N (EPI_ISL_1921288, EPI_ISL_1921764, EPI_ISL_1921765, EPI_ISL_1921768), and two isolates with 19R, 158R, 452K, and 681R (EPI_ISL_1921165, EPI_ISL_1921739), were collected on April 26, 2021.
