# Supplementary material for: Paired associated SARS-CoV-2 spike variable positions: a network analysis approach to emerging variants
Source: mSystems. 2023 Jul 11;8(4):e00440-23. doi: 10.1128/msystems.00440-23 (PMC10469592; doi:10.1128/msystems.00440-23)
Supplement: Table S3 — Exemplary 4×3 contingency table, one of the 14,365 R × C tables examined, showing the marginal (total row or total column), conditional (rows or columns), and joint (cells) distributions of the amino acid frequencies between functional positions 681 and 501 in the Greek data set of SARS-CoV-2 spike proteins. [file msystems.00440-23-s0009.docx]

**SI Table 3**. Exemplary 4×3 contingency table, one of the 14,365 r × c tables examined, showing the marginal (total row or total column), conditional (rows or columns) and joint (cells) distributions of the amino acid frequencies between functional positions 681 and 501 in the Greek dataset of SARS-CoV-2 spike proteins. The probability value in all three tests of independence for this table was ≤0.001, implying an association between specific combinations of residues at the two positions.

| \|  \|  \| **pos_501** \| \| \|  \| \| --- \| --- \| --- \| --- \| --- \| --- \| \|  \|  \| **N** \| **T** \| **Y** \| Total \| \| **pos_681** \| **H** \| *35* \| *0* \| *744* \| **779** \| \| **L** \| *2* \| *0* \| *0* \| **2** \| \| **P** \| *261* \| *1* \| *26* \| **288** \| \| **R** \| *9* \| *0* \| *0* \| **9** \| \|  \| Total \| **307** \| **1** \| **770** \| ***1078*** \| \|  \| \| \| \| \| \| |
| --- | --- | --- | --- | --- | --- | --- | --- | --- | --- | --- | --- | --- | --- | --- | --- | --- | --- | --- | --- | --- | --- | --- | --- | --- | --- | --- | --- | --- | --- | --- | --- | --- | --- | --- | --- | --- | --- | --- | --- | --- | --- | --- | --- | --- | --- |
